# PLC-Integrated Sensing Technology in Mountain Regions for Drone Landing Sites: Focusing on Software Technology

**DOI:** 10.3390/s18082693

**Published:** 2018-08-16

**Authors:** Jun-Ho Huh

**Affiliations:** Assistant Professor Department of Software, Catholic University of Pusan, Geumjeong-gu, 57 Oryundae-ro, Geumjeong-gu, Busan 46252, Korea; 72networks@pukyong.ac.kr or 72networks@cup.ac.kr; Tel.: +82-51-510-0662

**Keywords:** natural hazards, lightning, drone landing site, Internet of Things (IoT), artificial intelligence, AI, App, computer architecture, protocol

## Abstract

In the Republic of Korea, one of the most widely discussed subjects related to future logistics technology is the drone-based delivery (transportation) system. Much (around 75%) of Korea’s territory consists of mountainous areas; however, the costs of installing internet facilities for drone landing sites are very high compared to other countries. Therefore, this paper proposes the power-line communication (PLC) system introduced in the author’s previous study as an alternative solution. For the system design, a number of lightning rods are used together with a monitoring system. The system algorithm performs substantial data analysis. Also, as the author found that instantaneous high-voltage currents were a major cause of fire incidents, a three-phase three-wire connection was used for the installation of the lightning rods (Bipolar Conventional Air Terminal). Thus, based on the PLC technology, an artificial intelligence (AI) which avoids lightning strikes at the drone landing site by interworking with a closed-circuit television (CCTV) monitoring system when a drone flies over the mountain regions is proposed in this paper. The algorithm was implemented with C++ and Unity/C#, whereas the application for the part concerning the integrated sensing was developed with Java Android.

## 1. Introduction

Autonomous unmanned aerial vehicles (UAVs) [1], commonly called drones, are the focus of various industries for environmental and natural disaster monitoring, border surveillance, emergency assistance, search and rescue missions, and relay communications [2]. Also, small-sized multicopters are increasingly put into practice due to their convenience in deployment and their low acquisition and maintenance costs [3].

It is estimated that the aircrafts we commonly use are struck by lightning once or twice a year. As most civilian aircrafts usually fly at a high altitude between 6000 to 12,000 m, they are directly exposed to the danger of lightning, which normally strikes 30 to 100 times per second, and sometimes more than five million times a day throughout the world.

**Figure 1:**
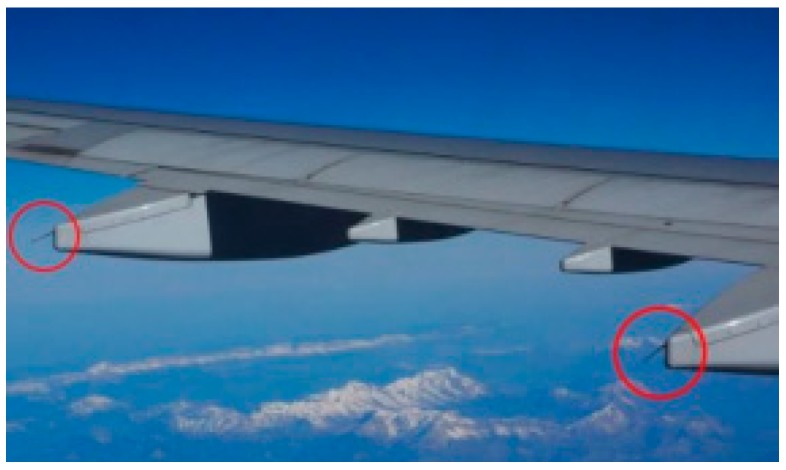
An aircraft can resume its flight safely because of the static dischargers (red circles) attached to its wings to discharge the electric current into the air.

A magnitude of electric force of about one billion volts and tens of thousands of amperes is generated when a plane gets hit by lightning, and, at such a scale, it can even burn it entirely. Although the plane’s instrument panel may shake or its surface may become sooty or slightly detached, this does not cause a serious problem inside the plane. Sometimes the passengers are not even aware of the incident. Nevertheless, after the plane lands, it must be checked thoroughly for any possible damage, possibly resulting in delays or cancellations of scheduled flights, causing travelers much inconvenience. Thus, what makes an aircraft safe against lightning? Acting as a large electric circuit, the plane instantly discharges the electric current into the air. The fuselage of an airplane is made of duralumin, an aluminum alloy with high conductivity. Figure 1 shows that an aircraft can resume its flight safely because of the static dischargers (red circles) attached to its wings to discharge the electric current into the air.

When a plane is struck by lightning, a strong current instantly spreads along the fuselage; however, a series of static dischargers attached to the wings discharge it into the air immediately. Acting as lightning rods, they can be seen from the windows close to the main or tail wings. There are several pointed parts under the wings ranging in size from 15 to 20 cm, and they are the static dischargers acting as lightning rods [4,5].

Static electricity can be generated when the electrons from the particles of dust, precipitation, or ice stick to the surface of a plane during flight or when the electrons on the surface are combined with these particles. Such static electricity should be dispersed into the air as it can cause a spark discharge at some sharp/pointed parts of the fuselage, or hinder communications.

The principle of dispersing a strong current from the plane is called the Faraday cage effect, which explains how a bird in a cage floating with electric current can survive. It is also the same principle that applies when one takes refuge in a car to be safe from lightning. The electric current is dispersed along the surface of the car body.

Meanwhile, although drones were originally developed for military purposes, they are now being used across a variety of industries including the entertainment, education, filming, and logistics sectors. The major players in these sectors are the United States (US) and China, as their vast territories constitute the optimal environment for drone operation. The Chinese company, DJI (Dá-Jiāng Innovations Science and Technology Co. Ltd), is one of the major players in the private sector and has already entered the Korean market. It is assumed that this company currently occupies about 70% of the global market share, and has an estimated value of around 10 billion US dollars. The Yano Research Institute forecasted that the worldwide drone market will expand to US$21.5 billion by the end of 2020, with the private sector contributing about US$8.7 billion of the total amount. Another consulting company, the Teal Group (US), expects that the global military drone market will increase to US$11.5 billion during the same period, while the United Kingdom (UK)’s PricewaterhouseCoopers (PwC) issued a positive forecast stating that the total economic value of the global drone market could reach up to US$130 billion [6].

The fast development of wireless sensor nodes led to a wider application of wireless sensor networks (WSNs) in the field of Internet of Things (IoT) systems which monitor or collect information from public service infrastructures, natural disaster relief works, the healthcare sector, smart homes, etc. At the same time, ubiquitous sensing that includes WSNs and other various applications provides substantial benefits over conventional networks and contributes to a novel perception definition of information management involving self-organization or distribution of information while achieving a low-cost operation. Although elements such as cost, power, topology, scalability, reliability, energy consumption, and operating environment may affect the design of a system, other factors should be considered as well [7,8,9]. On the other hand, computational intelligence (CI) is the fountainhead of artificial intelligence which often utilizes deep-learning, evolutionary algorithms as well as fuzzy logic. Some of the CI paradigms are being effectively used for tasks such as localization, optimal deployment, security, energy-aware routing and task scheduling, and data aggregation and fusion [10,11]. For the increasingly complex and dynamic environment of US, CI offers efficient and adaptive mechanisms that facilitate intelligent behaviors, offering better flexibility, autonomous behavior, robustness toward topology changes, communication failures, and rapid changes in the scenarios [12,13,14,15].

It is evident that both the US and China will play major roles in the drone market for many years to come; however, other countries are seeking opportunities in this market as well. The Japanese established a drone project center in the city of Chiba, near Tokyo, as one of their National Strategic Special Zones. The project involves the commercialization of a drone-based courier (delivery) service connecting major regional infrastructures throughout the country. The emergence of the drone industry is closely related to the Fourth Industrial Revolution, wherein various paradigms of hyper-connected IoT are bringing all existing worlds closer together. Drone technology and its infrastructure will also contribute to the development of sensors, artificial intelligence (AI), and other technologies of the future [16,17,18,19].

Despite the rapid growth of the drone industry, studies [20,21] pertaining to drone landing sites in mountainous areas and lightning avoidance systems are not performed enough by both major players. The Republic of Korea (ROK) is quite suitable for such research because of its landscape which makes it much costlier to install internet facilities in mountain areas to set up an efficient and convenient drone landing site. Another difficulty involved is the quality of the surge protectors used in the ROK. They usually create signal attenuations ranging between 30 dB to 60 dB when a power-line communication (PLC) signal band is used for communication. The problem is that the components of such a surge protector have some sort of self-capacitance element ranging from 1000 pF to 5000 pF, and a parallel condenser ranging from 0.1 uF to 5 uF, both of which could disrupt PLC. For this reason, it has become customary in the ROK to remove existing surge protectors when installing a lightning avoidance system. It is evident that such a practice makes the system more vulnerable to lightning strikes. Thus, based on PLC technology, this study proposes artificial intelligence that prevents the occurrence of lightning at drone landing sites in conjunction with a closed-circuit television (CCTV) monitoring system when a drone flies over a mountain region. The objective of this study was to introduce a PLC-based lightning protection system as an effective sensing and monitoring system. The issues pertaining to existing lightning rods and other components are discussed along with an efficient operational algorithm exclusively developed for the system.

The first lightning test conducted for drones at Manchester University revealed that drones are vulnerable to lightning; furthermore, external damage is greater when lightning conductors are used. Dr. Vidyadhar Peesapati and Dr. Richard Gardner joined with YouTuber Tom Scott to conduct a lightning test for the DJI Technology’s Phantom-3 drone and displayed it on YouTube [5]. In the first test, the drone struck by lightning instantly fell from the sky, and, in the second test where a lightning conductor was used, the damage was greater; the rotor became completely detached. For the test, they generated a stroke of lightning of over one million volts by using a 2 MV impulse generator in their high-voltage lab.

In the first test, the drone’s flight was simulated by loosely tying it in midair; however, when the lightning struck, it instantly fell. There was no evident external damage, but it was confirmed that all of the internal electronic devices were fried as the drone experienced electric resistance at least once. In slow motion, it was observed that a streak of lightning entered from one end.

For the second test, a lightning conductor was mounted to construct a more defensive mechanism against lightning. This was to investigate whether the lightning would normally bypass a quadcopter without causing any damage, as it does for airplanes. For the second drone, the experiment team arranged a piece of a conductive copper tape at the top as a lightning conductor.

After re-setting the lightning generator and delivering a stroke of lightning, the drone’s rotor broke off completely, causing greater damage than was observed in the first test, where there was internal damage only. This was due to the fact that the powerful force of lightning had blown the rotor before passing through the internal circuit. Considering that the inner structure was supposed to be protected, the result was worse than in the first test. Even though the result was unexpected, it still had some scientific significance. Progress was made for modern airplanes when static dischargers began to be installed on their wings based on research similar to the Manchester University experiment; however, this was the first research conducted on how drones would respond to lightning. In fact, a drone would not be flown on a stormy day as its battery life would be seriously shortened; however, as observed, the influence of weather is very large for drones.

The experiment team advised that it would be wise to refrain from flying a drone if there is a risk of lightning unless you are prepared to throw away thousands of dollars in an instant [4,5].

**Figure 2:**
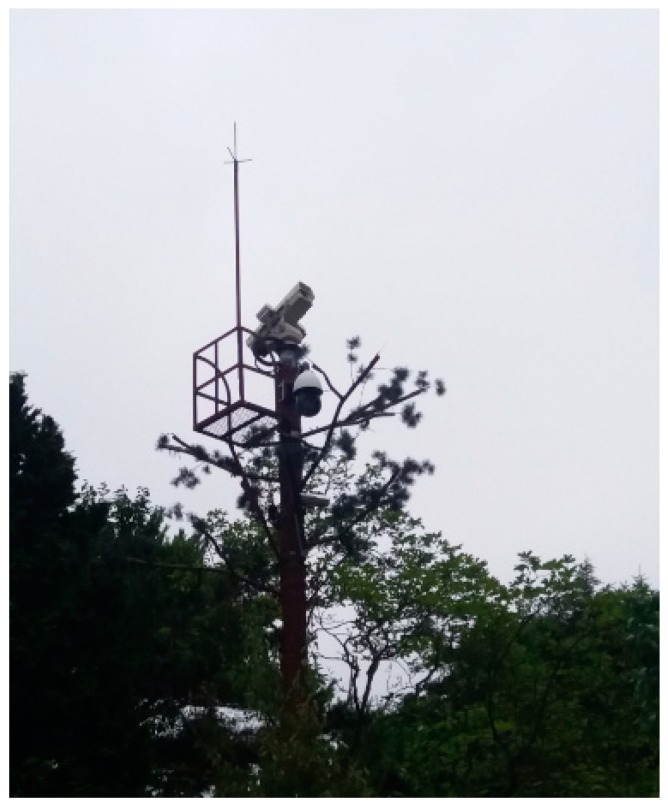
The power-line communication (PLC)-based lightning rod and intelligent closed-circuit television (CCTV) system installed at Beomeosa Temple (Geumjeong Mountain, Republic of Korea (ROK)) for a test bed experiment.

However, drone size and battery capacity are much different from those of airplanes. Also, due to the spontaneous nature of lightning and the fact that there are many lightning-prone regions in mountainous countries, it is not always easy to avoid lightning altogether. Although drones fly at low altitude according to the flight operation regulations of individual countries, they need to fly over lightning-prone regions in mountainous countries like the Republic of Korea (ROK) or Japan. Thus, a system which warns of a potential hazard by detecting lightning with lightning rods and CCTVs installed in mountain areas (Figure 2) is proposed in this study.

Additionally, an Android application was developed to allow drone operators to efficiently monitor flight status through a tablet or other portable device. The intelligent CCTV system can also be quite useful for routine forest fire surveillance.

## 2. Related Research

Unmanned aircraft systems (UAS), unmanned aerial vehicles (UAVs), and remotely piloted aircraft (RPA) are widely used by the military due to their potential in supporting various types of warfare.

The properties of these flight systems are required to be robust, adaptable, resource-efficient, scalable, cooperative, heterogeneous, and self-configurable. All such properties are viable as long as the physical control of individual UAVs is integrated with other factors such as navigation and communication capabilities. The algorithms and design principles proposed by research communities in the fields of wireless ad hoc and sensor networks, robotics, and swarm intelligence provided some valuable insights into the functionalities involved in the design of drones [3]. During the last two decades, there were several non-military development projects regarding UAVs (e.g., UAV-NET, COMETS, MDRONES, cDrones, OPARUS, AUGNet, RAVEN testbed, sFly, and MSUAV). The categories of these projects are as follows: (1) classification of vehicles depending on their size, payload, or flight time (e.g., helicopters, blimps, or fixed-wing UAVs). These elements actually affect factors such as the network lifetime, traveling distance, and communication range. (2) Classification depending on their design or algorithms. (3) Classification based on the type of application used [18]. The sophistication level of each application may vary depending on the objectives/requirements and the environment in which they will be used [19,20,21].

The development of drone operation algorithms based on computer science enhanced sensor-based navigation and image processing capabilities. Such development greatly improved the processing capability by handling a huge volume of information collected by the sensors. The process is required to be rapid, and the results must be accurate. Also, the algorithms must be autonomous and intelligent enough to comply with the objectives entered into their main memory.

As for the lightning protection system, the Bipolar Conventional Air Terminal produced by the Korean company, OMNILPS, is one that overcame the 260-year-old stranglehold of overseas air terminals. Taking advantage of the US approval of this product, it is expected that its introduction to major industries in the Republic of Korea (ROK) will increase. Some business sites of Samsung and Lotte groups are already replacing existing lightning rods with bipolar rods. Samsung studied the possibility of adopting bipolar rods for their high-precision production lines since 2009, and started installing them at its Hwaseong and Giheung factories, and recently extended them to other sites including its Pyeongtaek and Onyang factories. Similarly, the Lotte group installed them at the 123-story Jamsil Lotte World Tower, which is the company’s new landmark building. These cases are all based on the idea that bipolar rods will be able to protect their key production lines or properties from the ever-increasing number of lightning strikes due to serious global warming, while improving safety and reliability [10,22,23,24,25].

**Figure 3:**
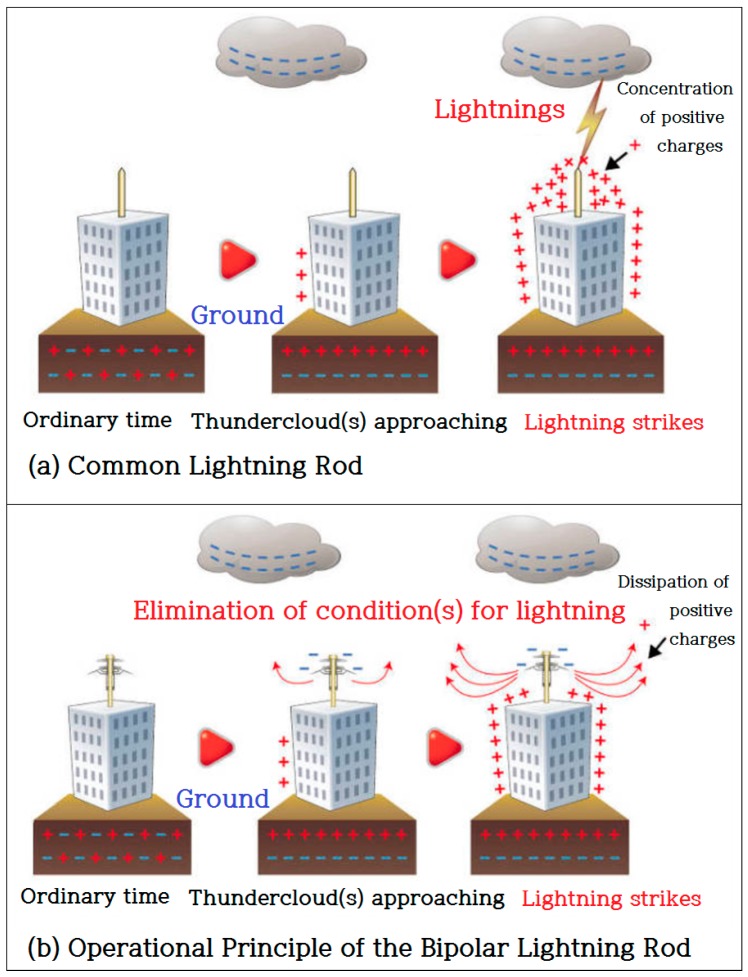
A common lightning rod and the operational principle of the bipolar lightning rod.

As shown in Figure 3, the reason for the success of bipolar lightning rods is that they surmount the problems with existing lightning rods. Common lightning rods discharge lightning currents on the ground via grounding following its down-conductor. The problem here is that surges could be generated in the equipment by electrostatic or electromagnetic inductions. Some sensitive electronic equipment at communication equipment or semiconductor production facilities is sometimes damaged by low-voltage surges in this process. On the other hand, bipolar rods discharge ground charges beforehand to avoid harmful situations. This is a fundamentally different process from other types of rods that induce lightning. Normally, when clouds with negative charges approach, positive charges gather around the main body of the lightning rod connected to the ground. At this point, the “discharging hat” insulated from the main body will have negative charges following the principle of bipolarity, such that the force of attraction between the positive charges on the main body and the negative charges on the hat increases until some corona discharges occur and the positive charges dissipate on the rods [10].

In the end, the principle is to basically shut out the activity of thunderclouds by transforming them into nonpolar clouds. Additionally, to achieve perfect protection, the surge protector device (SPD) is added to prevent damage by induced lightning, and, at the same time, the carbon ground rod is used to smoothly release currents into the ground. This technology can even prevent possible surge-oriented damages at source.

Meanwhile, deep neural networks achieved impressive success in fields ranging from object recognition to complex games such as Go [26,27]. Navigation, however, remains a substantial challenge for artificial agents, with deep neural networks trained by reinforcement learning [28,29,30] failing to rival the proficiency of mammalian spatial behavior, which is underpinned by grid cells in the entorhinal cortex [31,32].

Recently, the founder of Google DeepMind, Demis Hassabis, and his research team developed a path-finding AI [32]. There are some people who can find their way well if they have been down a particular road before. Others can find the destination with their animal-like instinct for direction. Now, an AI with such a path-finding ability was developed. As with the AlphaGo, the research team applied deep learning and reinforcement learning when they were developing the AI. This is similar to the network of grid cells which becomes activated when mammals recognize a space. Grid cells are referred to as “the global positioning system (GPS) in the brain” as they help animals find their way. The discovery of grid cells by May-Britt and Edvard Moser of the Norwegian University of Science and Technology earned them the Nobel Prize in Physiology or Medicine in 2014. Based on the results of their work, a new path-finding AI far better than existing ones was developed using a neural network possessing the characteristics of grid cells for reinforcement learning. The path-finding capability of this AI gradually improves by repeated learning. It adapts to a newly changed topography and finds shortcuts or exhibits much better skills than people in solving mazes [32].

The Google research team also used the AI to study brain functions. By comparing this AI with another version having no grid-cell neural network, it became evident that grid cells play a crucial role in estimating lineal distance or bearing when finding a path [32].

This study proposes a system that assists a drone in flying safely by detecting/avoiding lightning strikes with the aid of an AI-based CCTV monitoring system in the Korean topography where many low mountainous areas are present.

In terms of speed, it is more efficient to find an optimal route to fly safely, but it is not easy to avoid sporadic lightning strikes. The author plans on conducting research on the technology that automatically controls drones, which allows for judgments far superior to that of human beings. Delivering goods in mountain areas is still quite rare in the expansive landscapes of the US or China, but it could be quite effective in the ROK.

## 3. The Framework Design of the Korean Model PLC-Integrated Drone Landing Site in Mountain Regions

### 3.1. Path of Lightning and Cause of Damage

**Figure 4:**
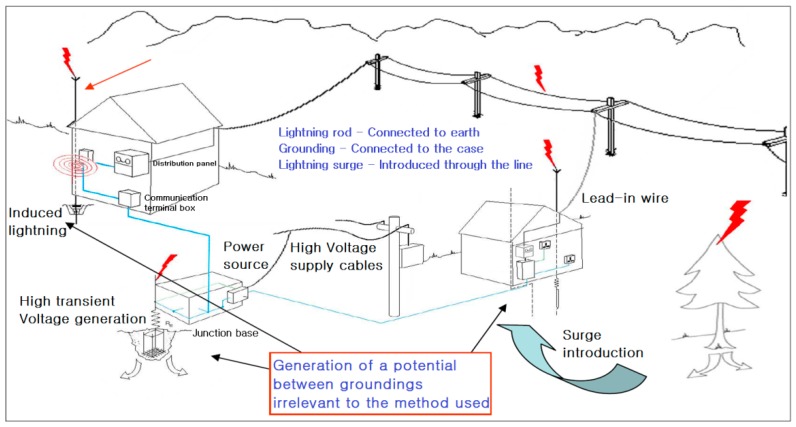
Path of lightning and cause of damage.

Figure 4 shows the path of lightning and damage caused by lightning. Even when the grounding is perfect, electrical surges through power, communication, and grounding lines can cause potentials between different lines. Lightning discharge and equipment damages occur at the weakest point of resistance.

**Figure 5:**
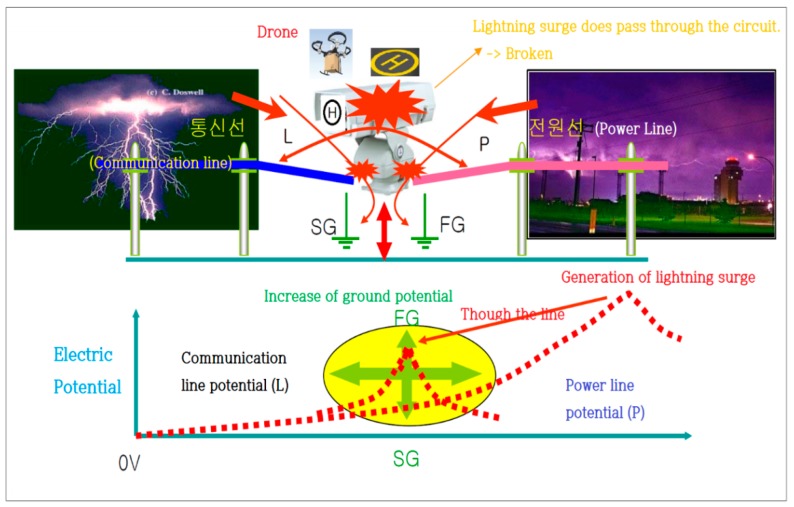
Penetration pathway.

When lightning is introduced through its penetration pathway, potentials between lines and groundings (L–SG & P–FG) occur, as well as between the lines (L–P). Damage is caused, firstly, by the discharged current through the circuit due to the difference in potentials between two different lines, and secondly, as SG forms a static coupling with the power (−) of the equipment’s circuit and the FG connected to the equipment’s outer box. The lightning surge from either line discharges current at the outer box (grounding) after passing through the circuit. Figure 5 shows the penetration pathway.

**Figure 6:**
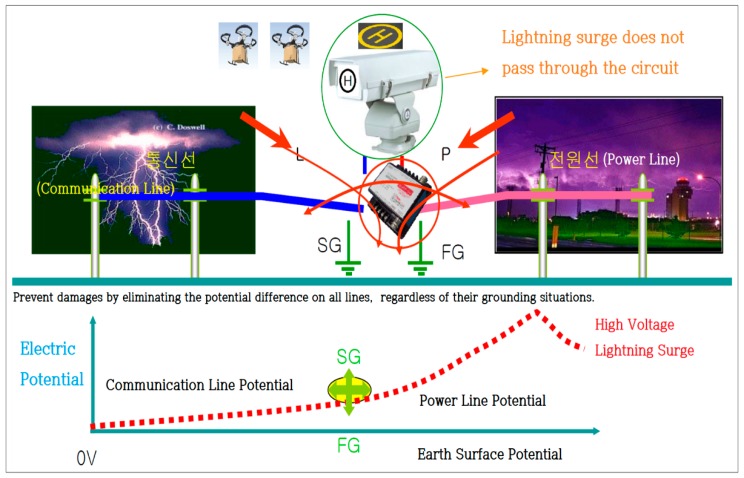
Principle of protection.

Figure 6 describes the protection principle, which safely protects the equipment from lightning surges by eliminating the potential difference between the lines.

Although lightning rods are essential for the protection of a building or structure from direct lightning, when considered in terms of equipment protection, they additionally introduce electrical surges existing around them, such that the better the performance of a lightning rod, the more surge currents will be introduced. The reason for this phenomenon is that a lightning rod induces electric charges that form in clouds to the ground, and these charges increase the ground potential, further increasing the potentials of the electric/electronic equipment through the grounding cable.

Increased ground potential causes potential with other points, and this potential flows backward from the grounding terminal, then passes through the circuit and propagates to other areas, ultimately causing damage. Thus, this paper proposes a lightning avoidance algorithm.

**Figure 7:**
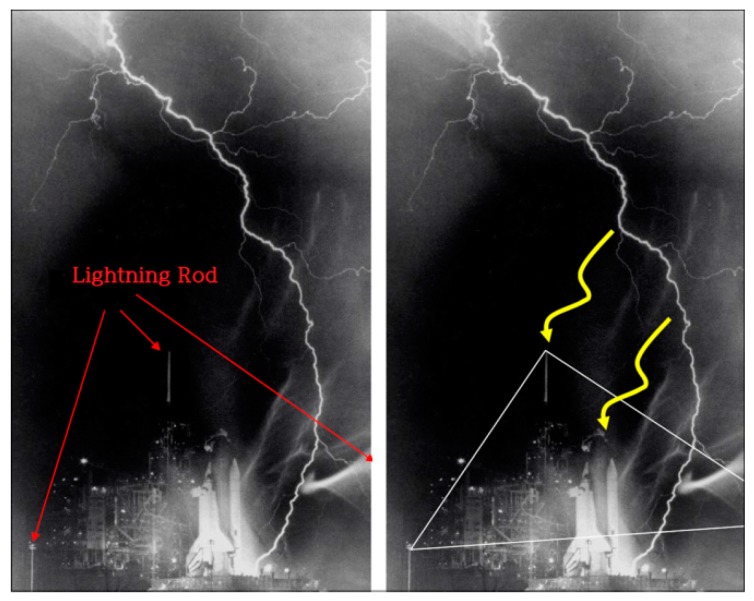
Installation of lightning rods and their problem.

Figure 7 shows an example of how lightning rods work, but also shows another case in which they fail. Considering such a case, this study suggests the use of AI, which can be used to make a decision on aborting the operation of a lightning-struck PLC station for 2 to 3 h when other stations are available.

### 3.2. The Framework Design

**Figure 8:**
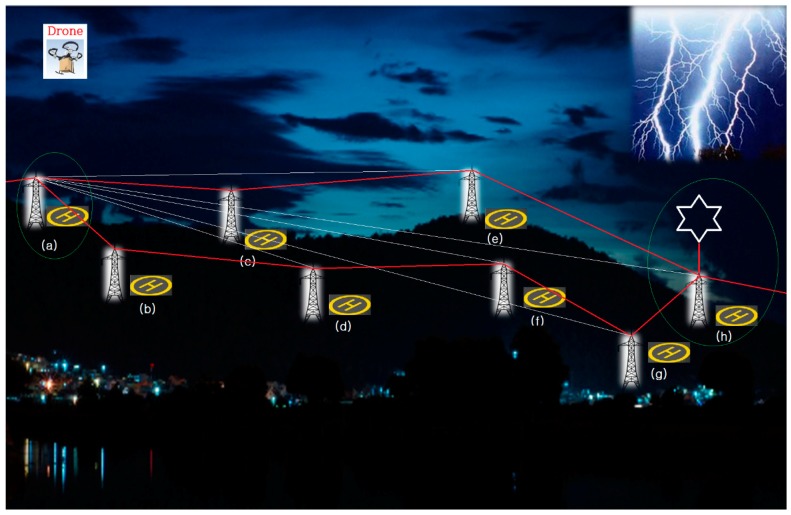
The framework design.

Figure 8 shows a bird’s-eye view to help the reader understand the system and the algorithm proposed herein. If lightning strikes the point (h), the voltages surrounding that area will be very high. Although most communication systems attempt to set priorities of communication based on distances following the logics of communication and networking engineering, the farthest communication system from the point (h) takes precedence over others whenever communications are interrupted or problems occur. As the ROK is a peninsula with many small mountains, a system with eight terminals that have communication ranges of 200 m to 2000 m (maximum PLC communication range) was constructed. This method was also used for the system proposed in the preceding study [2]. Actually, the three-phase, four-wiring method was previously used for a 220-V PLC system in a mountainous area; however, it made a lot of noise due to lightning strikes, additionally causing system breakdown and fire. The suggested configuration is designed to avoid such problems. In the ROK, there is a season during the summer, typically from June to July, in which PLC modems become moist. The company, SUNCOM [7,8], where the author once worked, developed a three-phase, three-wire modem with no grounding after conducting a series of researches aimed at eliminating or reducing the incidence of short-circuits and fire. Notably, navigating ships did not need grounding. Since grounding is essential in mountainous regions, a new type of lightning rod developed in the ROK was used in this study rather than an existing one. The author called this algorithm the “Lightning-Avoidance Algorithm”. In the event that lightning strikes (h), (a) will be used for communications instead of (e), (f), and (g) during a period of 2 to 3 h after the strike. The study also includes an intelligent system that allows a drone to change its flight route based on the lightning report for each operation station.

### 3.3. Proposed Method: Using C++

The algorithm was implemented with the C language. Proposed Algorithm (1) is an algorithm that excludes all edges and vertices within a certain range (p), taking the thunderstorm area as a criterion (vertex VL). The weight value of an edge is calculated by using the vertex VL and the vertex j connected to it. If the weight value stays within the range p, then the vertex j and edge VL–j are included in the “set of deleted vertices and edges”. Then, calculations are performed with the recursive call function for the edge and vertex connected to the same vertex; however, this time, the value of P will have a value obtained by deducting the weight of the previously deleted edge (P − weight of the deleted edge).

**Figure 9:**
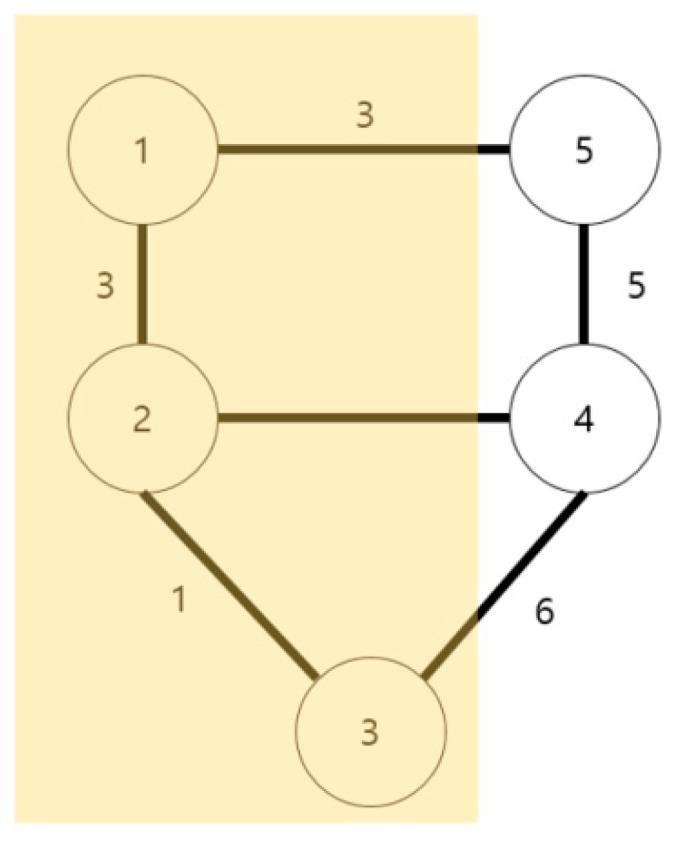
Proposed Algorithm (1).

Figure 9 shows the proposed Algorithm (1). Meanwhile, if P = 5 in Algorithm (1), the vertex and edge to be excluded from the relevant graph are indicated with a yellow box.

**Figure 10:**
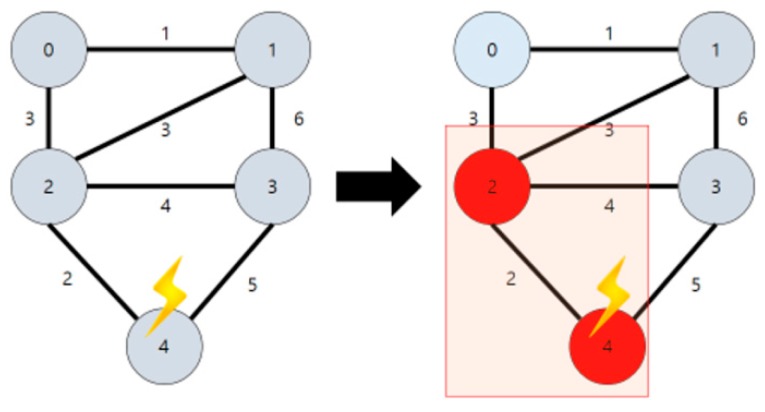
Proposed Algorithm (2).

Proposed Algorithm (2) is one that changes the currently created graph into a “minimum-cost spanning tree” (MST) using Kruskal’s algorithm. The vertex and edge to be added here will exclude the edge that contains the deleted vertex, which was calculated with Algorithm (1). Figure 10 shows the proposed Algorithm (2).

**Figure 11:**
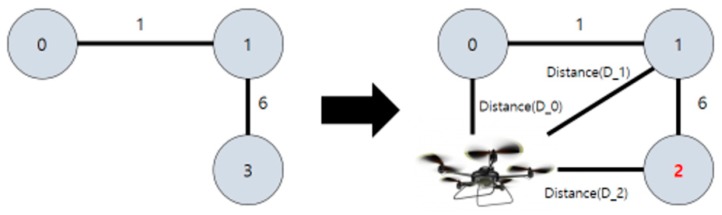
Proposed Algorithm (3).

Algorithm (3) renumbers the name of the filtered vertex and edge (diff = set of all vertices − set of all excluded vertices) and adds the distance (weight) between the drone and each vertex. That is, a graph is drawn once again using the vertices set diff, adding a vertex (drone) there, before distances between the added vertex and individual vertices of graph just created are calculated. Finally, the distances (weights) are added to the graph. With this graph, the shortest distance to each vertex is calculated with Dijkstra’s algorithm later on. Figure 11 shows proposed Algorithm (3).

Let us assume that a graph like the one shown above was created after analyzing a certain map. The thunderstorm area (vertex) can be identified using the above algorithms. Then, the graph such as that shown in the third picture can be reduced by excluding the vertices and edges included within the range P based on the vertex identified. Renumbering the edges and vertices in the reduced-scale graph creates a graph such as that in the fourth picture. Finally, adding a drone considering the position of it as a vertex, making the position as a starting point, the shortest distance to each destination can be output using Dijkstra’s algorithm.

## 4. Implementation of Proposed Method: Realization Using Unity/C# and Java

**Figure 12:**
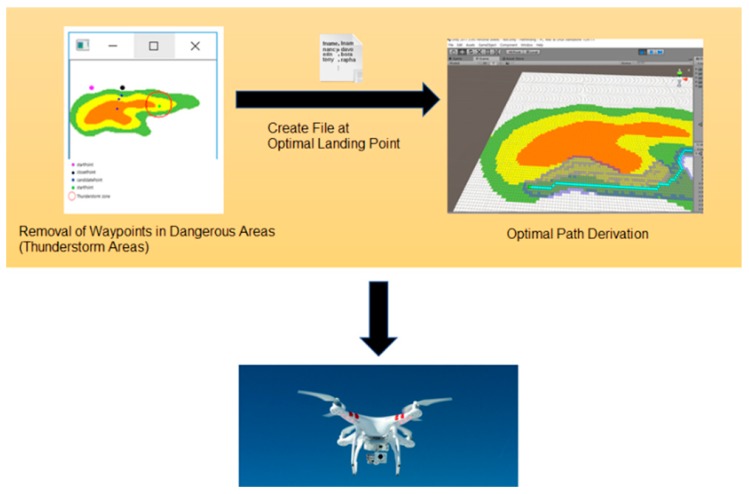
Aerial view of the thunderstorm area avoidance system.

As shown in Figure 12, when there are several arrival points in any area of mountainous terrain, there may be some distortion of communication between the arrival points located closest to the thunderstorm and the drone. Also, it is more difficult for a drone, as an ultra-light aircraft, to fly within the thunderstorm radius due to stronger winds. The following detail is an algorithm used to figure out the closest arrival point to the drone while eliminating the arrival point included in the thunderstorm radius. The first updated information in the drones after the thunderstorm is used to exclude the arrival points near the thunderstorm among the multiple arrival points.

It is an algorithm that draws all imaginary circles within a radius of 5 km (arbitrary value) around the point where the thunderstorm occurs, and then eliminates all possible landing points of the drones that may be within the circle’s range. The coordinate node of the arrival point is managed by the queue, and it is possible to estimate the distance by assuming that all landing points are known and by calculating the distance between the center of the thunderstorm. In other words, even if the drone moves stepwise or diagonally to the altitude in order to move to the arrival point, it takes three times longer to move vertically than horizontally. Each contour line in the map is of scale, as well as the landing point. When viewing the map on a coordinate plane, the distance can be figured out if the coordinates of each point are known.

The arrival points that are not included in the thunderstorm’s radius are placed as candidate points. In fact, this is a way of finding the nearest destination point of the current drones among the candidate points by the length of the hypotenuse using the Pythagorean theorem. In Figure 12, each point forms a triangle with the starting point, and the length of the hypotenuse is the length reflecting the straight line distance (base) and the height of the arrival point, which enables one to know the distance between the drone (which can be moved horizontally and vertically) and the arrival point.

**Figure 13:**
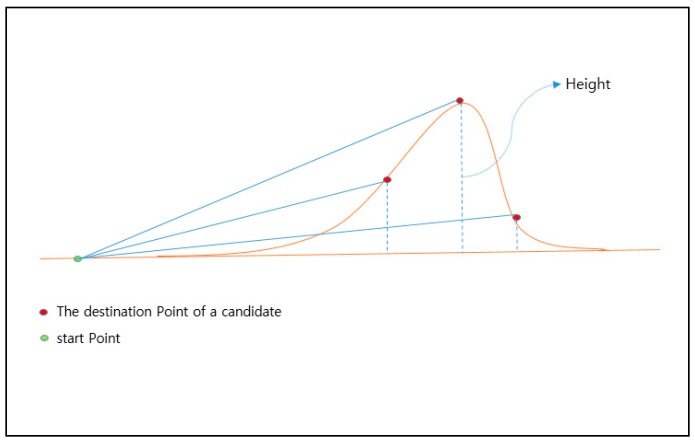
Inducing the shortest distance.

In a normal case, however, a cross-section of the mountain as viewed from the side is not easy to obtain, as shown in Figure 12, but the height of the arrival point can be figured out through the contour of the mountain. If the distance (base) between the height and the starting point can be known using the Pythagorean theorem, it is not difficult to determine the length of the hypotenuse. Figure 13 shows an example of inducing the shortest distance from the mountain’s cross-section.

The important point here is the weight of the height. The height must be at least three times the weight of the straight line, because most drones are about three times faster than their vertical speeds. A ratio of 1:25,000 represents an altitude difference of 10 m. Thus, it has a length of 30 (10 (height) × 3 (magnification)), 60, 90, etc. according to the height of the contour line.

**Figure 14:**
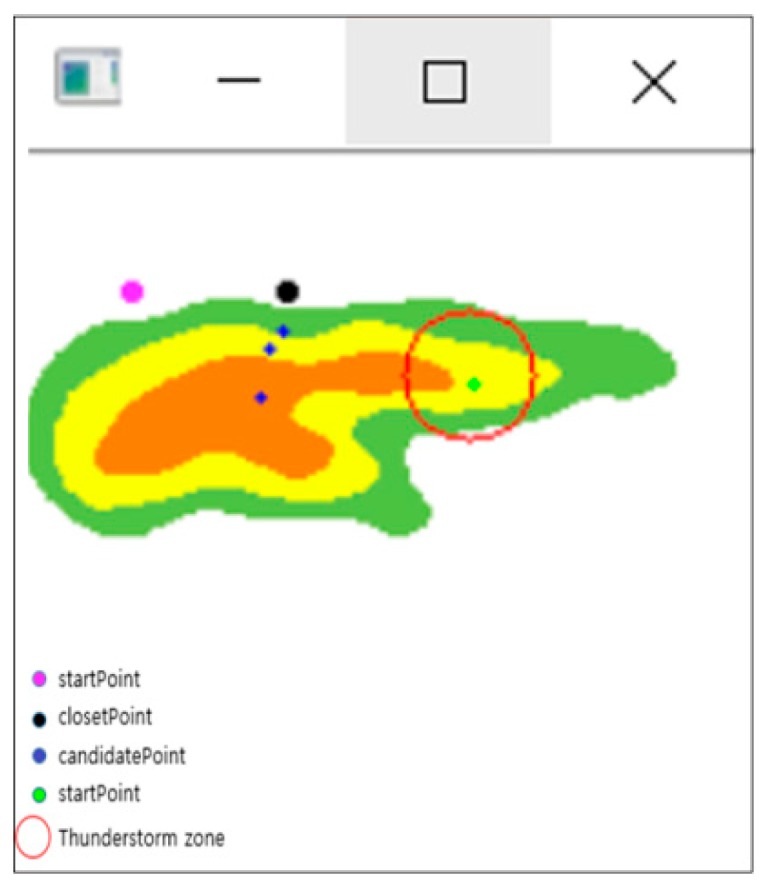
Each point expressed on the contour.

Figure 14 shows an initial screen with one starting point and five arrival points. It assumes that the red circle is a thunderstorm area for calculating the distance between the arrival points using Algorithm (1), and displays the existing points within the range in green. Arrival points that do not belong within the thunderstorm range become candidates.

**Figure 15:**
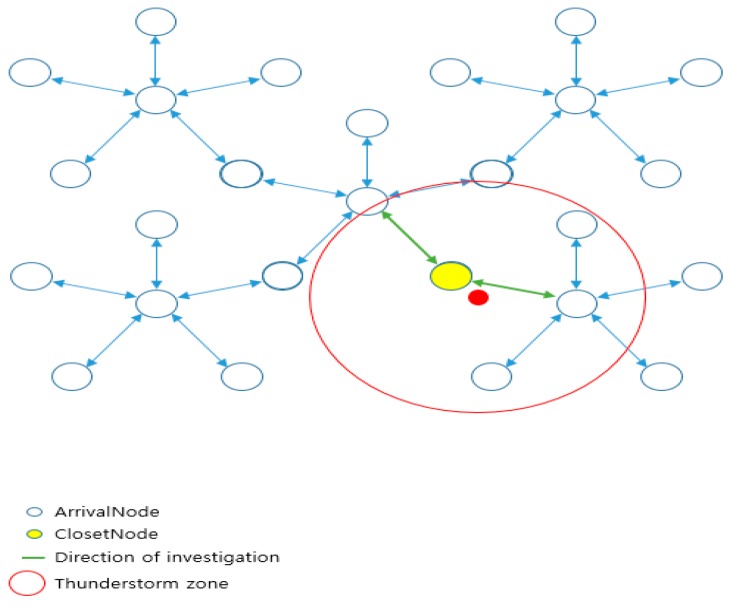
Node structure of points installed at regular intervals.

Figure 15 shows the node structure of points installed at regular intervals. There may be better algorithms, but it is not easy to determine which strategy to use in order to install the arrival point since the drone’s autonomous navigation system is yet to be commercialized. Thus, if it is necessary to investigate all nearby destinations, and if it results in more efficient management and time complexity, the arrival points can be managed in groups by the area, reducing the number of n. For the installation of strategic arrival points, however, a better algorithm can be implemented if it is installed irregularly using random point installation rather than being adjacent to each other at regular intervals. In this case, if each arrival point is nodalized and the adjacent nodes are connected, it is possible to find the nearest node in the thunderstorm area. Also, only the neighboring nodes of the thunderstorm area are examined to realize a better algorithm. If the order of the nodes is aligned according to the coordinate values when finding the closest node, the closest node to the thunderstorm can be efficiently found.

The A* algorithm can induce the route to the destination in a relatively short period of time without searching for many peaks, unlike the commonly known Dijkstra algorithm. However, it is not suitable for application to Korea’s territory, which consists of many mountainous areas. In this implementation, the shortest distance is induced by using the Dijkstra algorithm, and it also compares the results with the final A* algorithm, which was improved to make it suitable for Korea’s mountainous terrain.

Since high-altitude, unmanned aerial vehicles perform missions at over 9000 m on average, there is no exception to altitude; however, drones belonging to the class of low-altitude, unmanned aerial vehicles perform missions at altitudes below 6000 m, which makes it difficult to obtain the results desired by users. If it is possible to determine the location of the landing point and select the rate of altitude change, it is possible to identify the optimal route.

A definition of the graph object is responsible for data output related to the graph using the distance calculation in the graph composed of node data and movement function to the adjacent node. Vector 2 is an object for two-dimensional motion information, and is stored in the “allDirections” list to move eight directions from the current position.

Calculating the shortest distance between the starting point and arrival point on pixel can be done using Equation (1).

**Figure 16:**
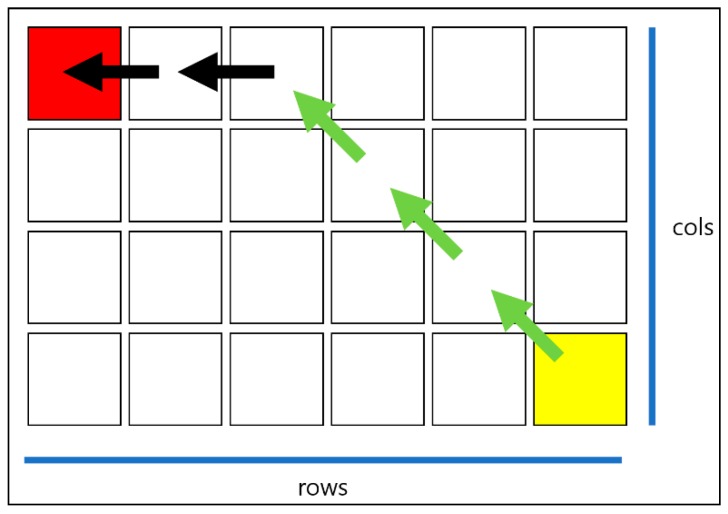
Example of distance between the starting point and arrival point on the map.

**Figure 17:**
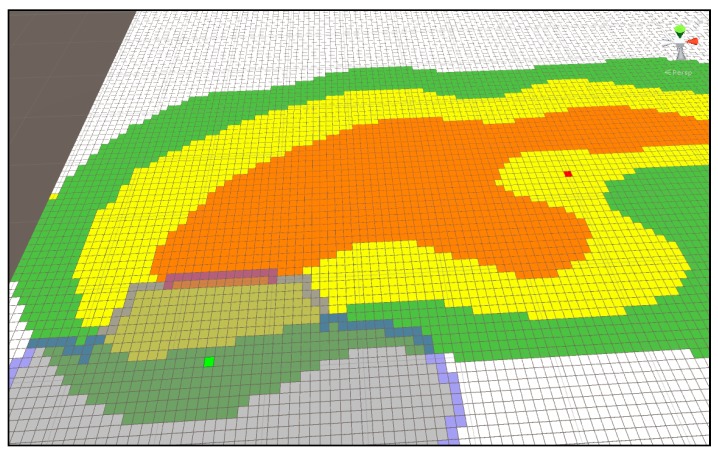
Search process of multi-extra.

When calculating Equation (1) using Figure 16, the total diagonal distance on the pixel becomes the smallest value of 4, which is 6, of the two rows and columns, while the distance of the straight line becomes 2. The shortest distance between the starting point and the arrival point of Figure 16 value of 4 is 8 (including the destination). Also, Figure 17 is an example illustrating Equation (1), and these two illustrate “GetNodeDistance”. In this program, it is assumed that each node maintains 1 × 1 form. The diagonal distance per node is 1.5.

(1) Diagonal numbers=min(rows, cols);Straight numbers=max(rows, cols)−Diagonal numbers.

**Figure 18:**
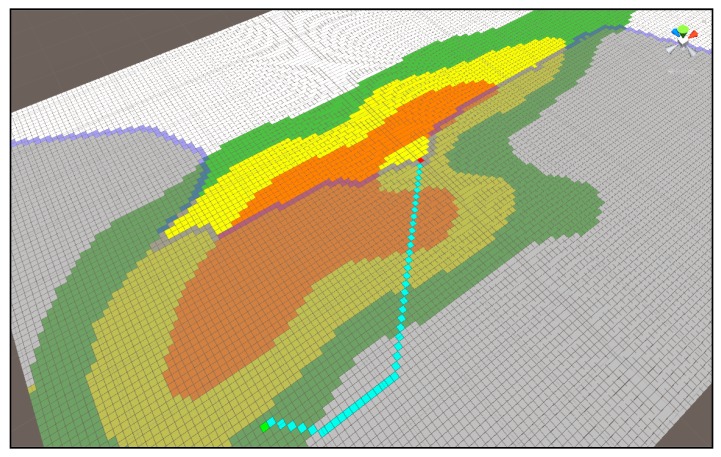
Shortest distance display using multi-extra.

Figure 18 shows the shortest distance display using multi-extra. It proceeds in a spreading form at the green starting point and continues until the search area reaches the arrival point. The map represents the mountainous terrain, and each color in the contour line represents the height. Green is the lowest, then yellow, and the highest area is orange. In the search process, Dijkstra considers the weight on each height and seeks the shortest route. When an arrival point is found, it enters by referring from the previously found node until the starting point of the previous node is found. In this way, it displays the route while riding.

Figure 18 is the result for Figure 17, and the blue pixel represents the shortest distance from the starting point to the arrival point with the height of the mountainous terrain as a weight.

**Figure 19:**
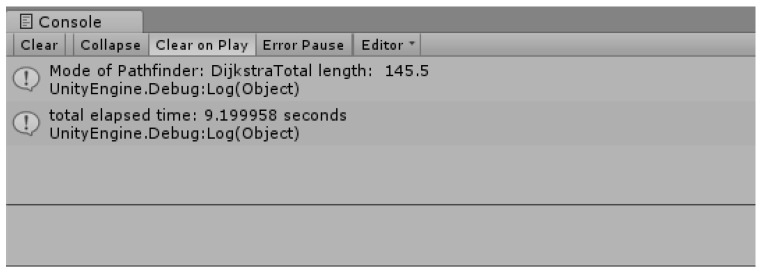
The shortest route length and the travel time.

Figure 19 shows the time taken for the search and the length of the shortest route. Although it is called the Dijkstra algorithm using the priority queue, it takes a lot of time to search, and the length of the shortest route is longer because the height of the current drone (the height of the current terrain) is not considered relatively.

If the node type of the current node is checked and the type of neighboring node is the same, “−1” is added to compensate the error caused by computing the data in float format.

**Figure 20:**
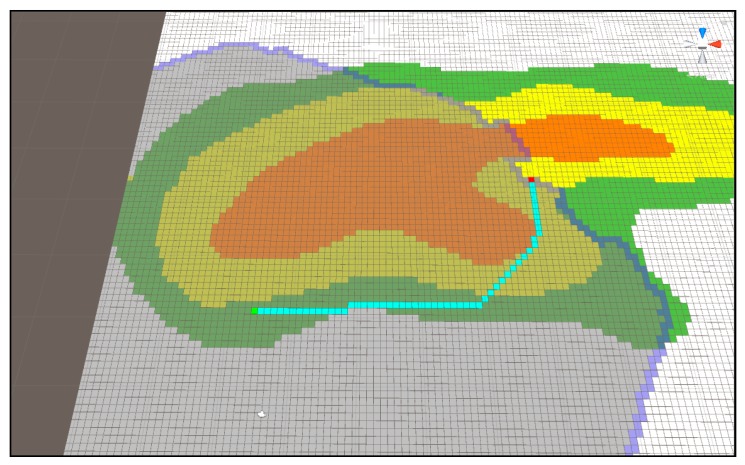
Shortest distance search result of improved Dijkstra Algorithm (1).

Figure 20 is the result of an improved Dijkstra algorithm with the addition of Algorithm (1), and Figure 18 shows the difference in the area width of the route and search.

**Figure 21:**
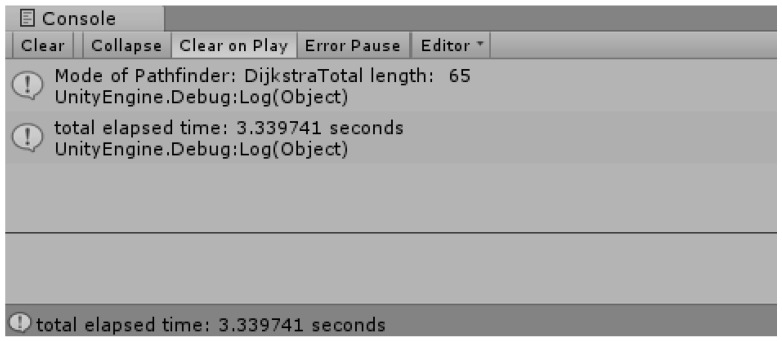
Shortest distance search result of improved Dijkstra Algorithm (2).

Figure 21 is a data version of the result of the improved Dijkstra algorithm. Unlike Figure 19, the length of the route was improved from 145 to 65 (about two-fold), and the time taken for the route search also improved three-fold from 9.19 s to 3.33 s.

The program adds two functions to the topography of the mountainous terrain of Korea. The first is to consider the shortest distance including the altitude (weight), and the second is to find the shortest distance while keeping the current altitude (the height of the current terrain).

It adds the part of the improved Dijkstra algorithm [24] implemented previously, and introduces the concept of relative distance. That is to say, it is a method of subtracting only the difference of the vertices of neighboring nodes. If the height of the current location is the same as the height of the neighboring location, the added weight is not specified. It can be applied very efficiently to high-altitude sections. However, it may not be suitable for the zone with a relatively high uphill section of the mountain terrain, where the landing point is located at approximately the same height as the drone, but it needs to detour in the middle due to mountains.

**Figure 22:**
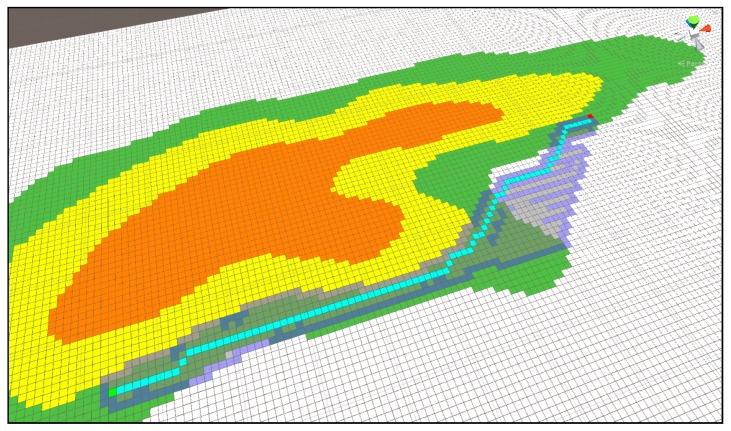
The route that takes the shortest distance using Dijkstra Algorithm (1).

**Figure 23:**
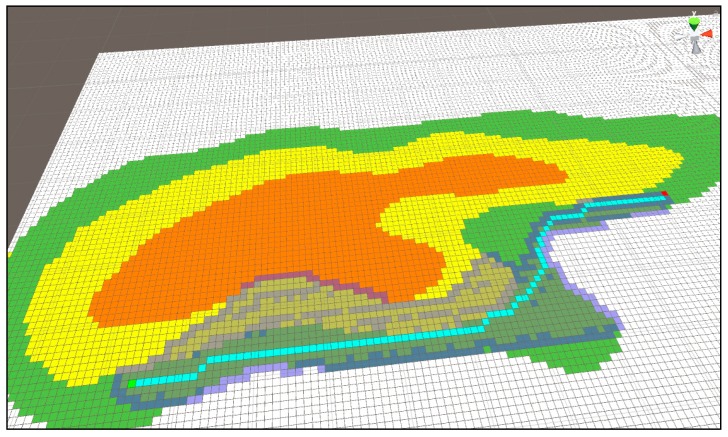
The shortest distance search method while keeping the current terrain as far as possible using Dijkstra Algorithm (2).

Figure 22 and Figure 23 show the route difference when applying Algorithm (1) and (2) to the A* algorithm using the same starting point and arrival point. Unlike Figure 22, Figure 23 shows a tendency to keep the current terrain. The time necessary to search these paths and the length of the output path are outlined below.

**Figure 24:**
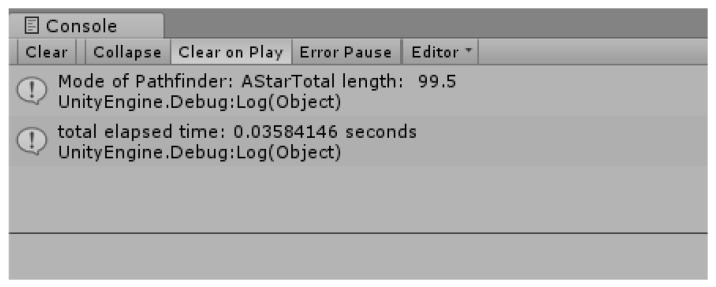
Result using Dijkstra Algorithm (1).

Figure 24 shows the time taken to obtain the results of Figure 22 and the length of the shortest path. The time required to find a path and the areas searched for in the existing A* algorithm are much smaller than those of the previous Dijkstra algorithm, and the path is also found to be relatively optimal.

**Figure 25:**
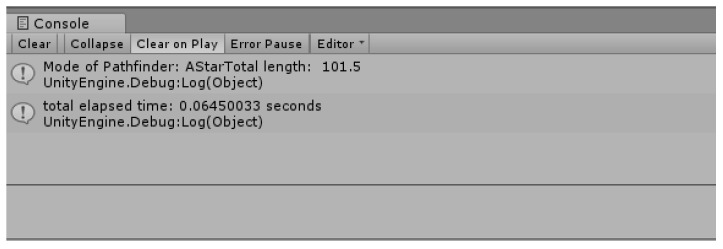
Result using Dijkstra Algorithm (2).

Figure 25 is time taken and path length to obtain the result in Figure 16. As it has a strong tendency to return, it takes a longer route and time compared to Figure 24. This program enables toggling the algorithms Dijkstra Algorithm (1) and Dijkstra Algorithm (2), inducing the optimal route of the landing point where no thunderstorm affects the drone according to the characteristics of the terrain, while interlocking with the program that outputs the thunderstorm area avoidance and landing coordinate data.

## 5. Implementation of Proposed Method: Realization Using Java and Java Android

Many drone products already have the function of moving drones according to the waypoints. This function sets the altitude according to topographic data in Google Earth. Since the altitude is fixed at the maximum height, it does not provide an optimized path.

**Figure 26:**
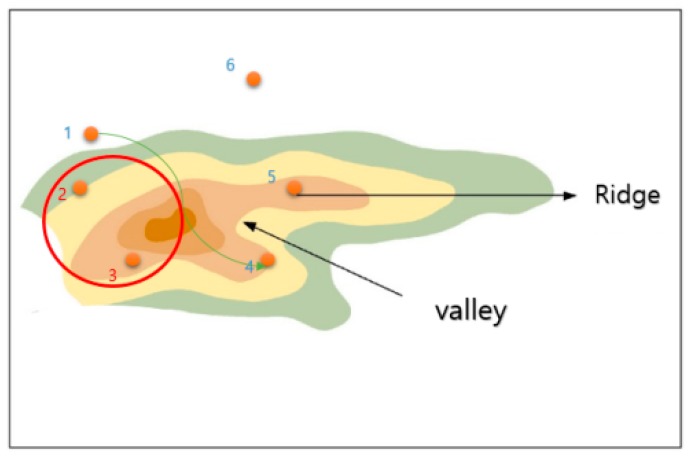
Progress path according to the waypoint expressed in OpenCV.

In Figure 26, there may occur a case that requires revision in the defined order of waypoints due to sudden natural disasters (thunderstorms, strong winds, etc.) while moving along waypoints. Assuming that the red circle is considered as a dangerous area in Figure 26, it is necessary to go directly to the next area passing through the waypoint included in the area. It is also necessary for the drone to bypass safely without passing through the area.

**Figure 27:**
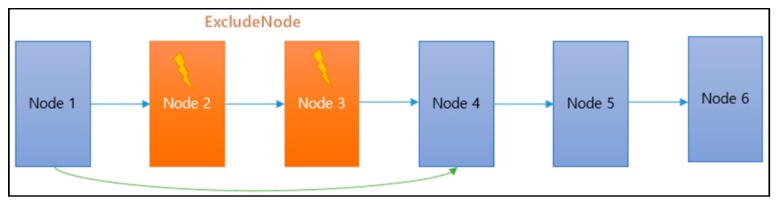
Waypoint node that removes the dangerous area.

Figure 27 is a diagram describing the waypoint path revision as a node structure that is described in Figure 26. Each node is the arrival point of the drone. Nodes 2 and 3 are determined as the arrival points included in the dangerous area. Thus, the path is removed from the waypoint path, and the path is immediately changed from the first node to the fourth node.

The distance to the center point of the dangerous area is calculated at each node. If it is included in the range, it is removed from the queue and the color of the point is changed for readability (“DrawCircle”).

The proposed code assumes a range of danger zones by drawing a circle with a radius of 5 km from the central area, but the entire adjacent sector nodes listed below can be classified as a dangerous area. After this process, the path is modified to Node 4 from Node 1 skipping Node 2 and Node 3. If the waypoint path is closer to the straight line, however, the distance from Node 1 to Node 4 becomes longer, which increases the burden of the drone. Considering the performance of currently available drones, a solution is needed for this problem.

Among the solutions presented at short flight speeds regarding embedded technology, many of the methods discussed in the literature simply install more points so that the battery can be charged during the flight if there is a need to charge the battery. If there are many points, the objective is to find the safe point (other than dangerous area) located closest.

**Figure 28:**
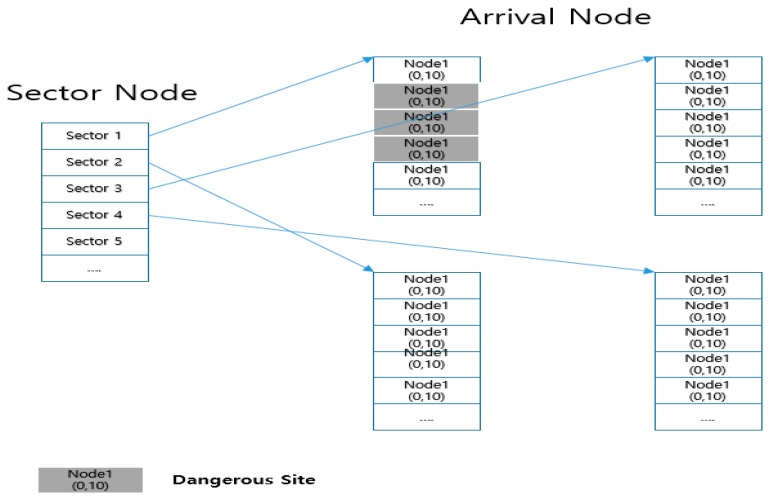
Node-tree structure of waypoints (arrival nodes).

Figure 28 shows the node-tree structure of the waypoints (arrival nodes). It is not efficient to search all the waypoint nodes to find the closest node in order to calculate the distance from the drone’s position. It is also difficult to estimate the number of points if they are irregularly scattered on a large map. So, this is a method to place all nodes into a sector node. In each sector node, arrival nodes (waypoints) included in the sector range are stored, which enlarges the range of nodes to be searched. Attention must be paid that the removed node is considered to be a dangerous area and is excluded from the adjacent node candidates to access.

**Figure 29:**
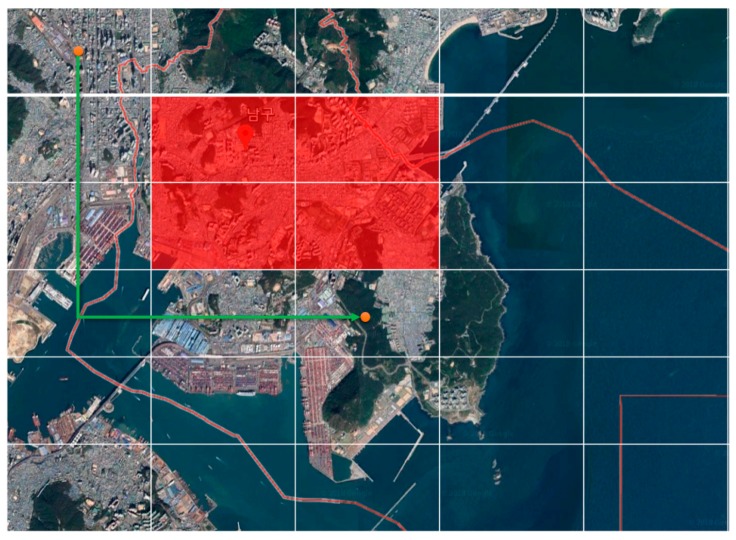
Sectoring the area expressed in the map provided by Google Earth.

Figure 29 shows sectoring the area expressed in the map provided by Google Earth. The code in Figure 27 assumes a radius of danger, but it may expand the entire sector adjacent to the dangerous area as a dangerous area. If so, node candidates excluded in the search for the closest node are not excluded from the arrival node (waypoint node), but they are excluded from the sector node. Access to the relevant sector is restricted for the drone not to pass through when moving toward the next node.

If it does not hit the range of map (wall) where the 8-direction is managed, it becomes the search target sector. Since the distance between two points is given by the pixel coordinate values, x and y, it can easily be calculated using the Pythagorean theorem which finds the length of the diagonal line. The coordinates of the shortest node at the found distance are stored in an independent node called “closetNode”.

**Figure 30:**
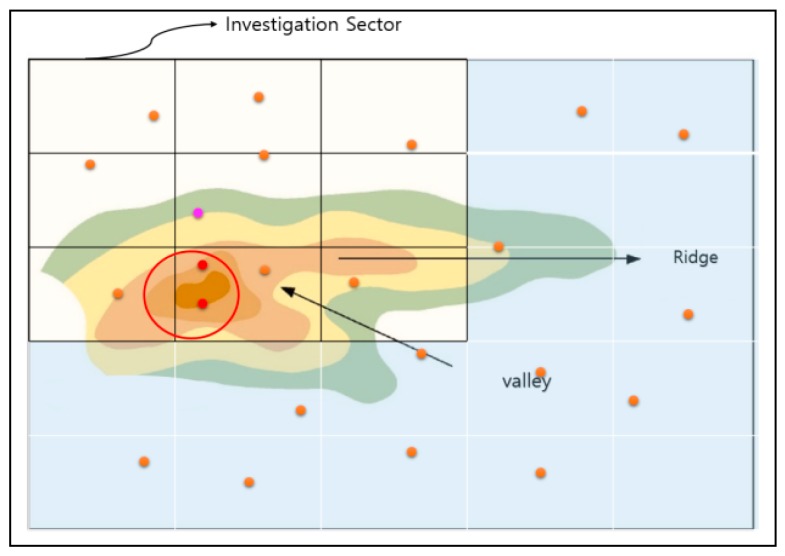
Sector subject to search according to the sectoring waypoint.

Figure 30 shows the sector subject to the search according to the sectoring waypoint. It is possible to search the closest node more efficiently than the method of calculating the position between all existing waypoints and drones. If there are more waypoints on the map, and if a larger map is used, it performs better. The arrival node (waypoint node) is reduced for a distance calculation since it is necessary to search the sector nodes located in the 8-direction only.

**Figure 31:**
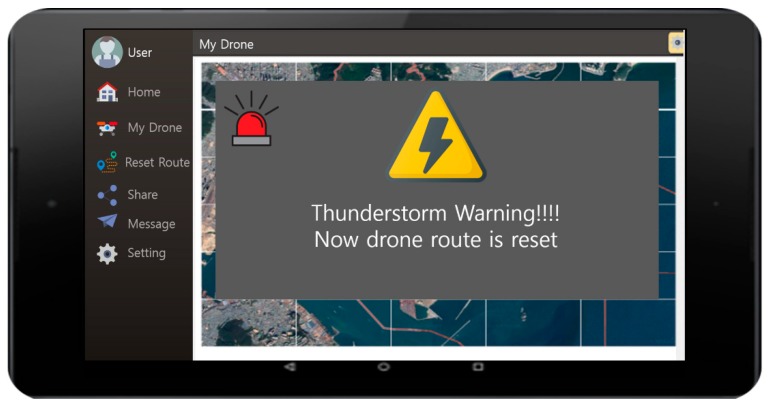
User interface showing the lightning/thunderstorm warning screen.

Figure 31 show the user interface used to warn lightning strikes. The menu on the left side of the screen shows Home, My Drone, Reset Rout, Share, Message, and Setting. The drone operation and its current status can be managed with a smartphone or a tablet mounted with an Android OS. The screen also shows the warning message in the case where there was a lightning strike on the drone’s current flight path. Following the warning, the message indicates that the drone’s route is reset. The screen after the message will show the user the information regarding the drone’s original route together with its new route planned due to the lightning strike.

**Figure 32:**
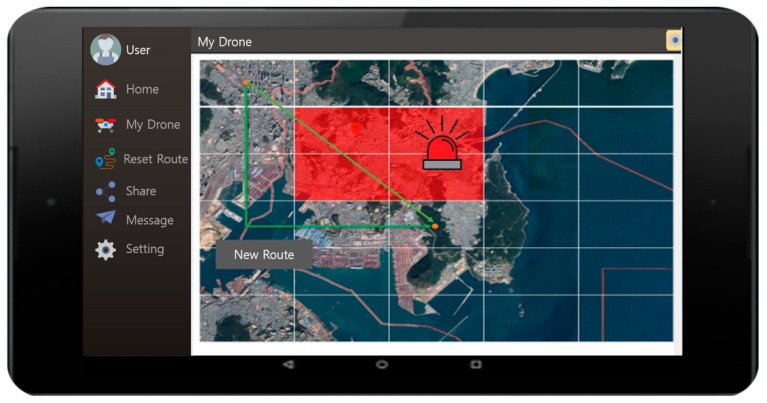
The lightning danger area and the recommended route (reset).

Figure 32 shows the lightning danger area and the route that was reset. The zones on the map are divided, and the lightning areas are indicated in red. The light-green line is the original route and the dark-green line is the new recommended route. With this user interface, the drone is able to avoid the danger zone and follow the new route to be safe.

## 6. Discussion

When delivering goods, delivery drones in large continental countries like the US or China are seldom exposed to bad weather as they can avoid mountainous areas. However, nations covered with many mountainous regions provide a dangerous environment for drones, especially with regards to thunder and lightning. Thus, an AI system which intelligently detects lightning spots and informs them to a drone to avoid the hazardous area and to find an optimal landing site was developed and proposed in this study. This study includes an intelligent system which allows a UAV to change its route based on the report on the lightning areas.

## 7. Conclusions and Future Work

Because of the signal attenuations caused by surge protectors that affect PLC bands, PLC systems are often set up without it. As the self-capacitive characteristics of the components used for surge protectors and the parallel condenser used to remove noise are the main causes of attenuation, PLC systems built in mountainous areas are often vulnerable to lightning strikes and electrical surges. Thus, the author of this paper proposes a lightning avoidance design that adopts lightning rods produced with an effective shock-resistant material together with the monitoring system surrounding them. The system algorithm was developed with C++ and Unity/C#. Also, the drone operation and its current status can be managed with a smartphone or a tablet mounted with an Android OS.

The algorithm performs substantial data analysis in order to design an efficient framework for a drone landing site. Recognizing that many fire incidents were caused by instantaneous high-voltage currents in the past, a three-phase, three-wiring connection was adopted for this system.

Thus, based on PLC technology, an artificial intelligence that avoids lightning at the drone landing site by interworking with a CCTV monitoring system when a drone flies over the mountain regions was proposed in this study.

The main contribution of this study concerns the introduction of a new drone guidance system in mountainous areas, in which AI is used to guide the drone to avoid unsuitable landing sites and to select the most suitable landing site by informing it of possible lightning points based on the intelligent observation of the environments of potential landing sites. The study also includes an intelligent system that allows a drone to change its flight route based on the lightning report for each operation station. As PLC technology and its applications were proposed and implemented in many systems around the globe in the past, another objective of this study was to present a more efficient algorithm for the processing of sensor-collected data. Intelligent and autonomous data-processing capability is still a major issue in drone technology, and related studies are being conducted continuously. Although this research focused on the software aspect of the drone and lightning protection/avoidance system, it is true that hardware such as sensors, lightning rods, and other mechanical components should be improved as well to provide better performance. This research will be expanded by the author in future studies by including more simulations and comparisons with existing approaches. One of the limitations expected for such a study is that drone technology in the ROK is still at an early stage compared to the other major players; thus, the research data may be insufficient.
